# Identification of Multiple Pancreatic and Extra-Pancreatic Pathways Underlying the Glucose-Lowering Actions of *Acacia arabica* Bark in Type-2 Diabetes and Isolation of Active Phytoconstituents

**DOI:** 10.3390/plants10061190

**Published:** 2021-06-11

**Authors:** Prawej Ansari, Peter R. Flatt, Patrick Harriott, J. M. A. Hannan, Yasser H. A. Abdel-Wahab

**Affiliations:** 1 School of Biomedical Sciences, Ulster University, Coleraine BT52 1SA, UK; pr.flatt@ulster.ac.uk (P.R.F.); p.harriott@ulster.ac.uk (P.H.); y.abdel-wahab@ulster.ac.uk (Y.H.A.A.-W.); 2 Department of Pharmacy, Independent University, Dhaka 1229, Bangladesh; jmahannan@iub.edu.bd

**Keywords:** glucose, insulin, obesity, diabetes, DPP-IV, islets, beta cells, phytochemistry

## Abstract

*Acacia* *arabica* is used traditionally to treat a variety of ailments, including diabetes. This study elucidated the antidiabetic actions of *A. arabica* bark together with the isolation of bioactive molecules. Insulin secretion and signal transduction were measured using clonal β cells and mouse islets. Glucose uptake was assessed using 3T3-L1 adipocytes, and in vitro systems assessed additional glucose-lowering actions. High-fat-fed (HFF) obese rats were used for in vivo evaluation, and phytoconstituents were isolated and characterised by RP-HPLC followed by LC-MS and NMR. Hot-water extract of *A. arabica* (HWAA) increased insulin release from clonal β cells and mouse islets by 1.3–6.8-fold and 1.6–3.2-fold, respectively. Diazoxide, verapamil and calcium-free conditions decreased insulin-secretory activity by 30–42%. In contrast, isobutylmethylxanthine (IBMX), tolbutamide and 30 mM KCl potentiated the insulin-secretory effects. The mechanism of actions of HWAA involved membrane depolarisation and elevation of intracellular Ca^2+^ together with an increase in glucose uptake by 3T3-L1 adipocytes, inhibition of starch digestion, glucose diffusion, dipeptidyl peptidase-IV (DPP-IV) enzyme activity and protein glycation. Acute HWAA administration (250 mg/5 mL/kg) enhanced glucose tolerance and plasma insulin in HFF obese rats. Administration of HWAA (250 mg/5 mL/kg) for 9 days improved glucose homeostasis and β-cell functions, thereby improving glycaemic control, and circulating insulin. Isolated phytoconstituents, including quercetin and kaempferol, increased insulin secretion in vitro and improved glucose tolerance. The results indicate that HWAA has the potential to treat type 2 diabetes as a dietary supplement or as a source of antidiabetic agents, including quercetin and kaempferol.

## 1. Introduction

Diabetes mellitus (DM) is one of the fastest-growing metabolic disorders resulting from deficiency of insulin, disturbed beta-cell function or insulin resistance [1]. Type 1 diabetes mellitus (T1DM) typically develops under the age of 30, when beta cells are destroyed. Type 2 diabetes mellitus (T2DM) is commonly found over the age of 40, but it is increasingly common in children and young adults due to childhood obesity, which causes beta-cell dysfunction and insulin resistance. Both major forms of diabetes are characterised by hyperglycaemia, which is a major player in the risk of developing diabetic complications such as cardiovascular disease, neuropathy, retinopathy and nephropathy [2]. Diet, weight loss and the use of single or multiple oral antidiabetic drugs are prevalent treatment options for T2DM. Such agents, including DPP-IV inhibitors and glucagon-like peptide-1 mimetics, are being used to improve glucose tolerance via the potentiation of glucose-stimulated insulin secretion [2,3]. GIP and GLP-1, two incretin hormones secreted from intestinal K and L cells after a meal, are very effective regulators of postprandial hyperglycaemia [4]. The DPP-IV enzyme dictates the half-life of incretin hormones by cleaving the first two N-terminal amino acids to generate inactive forms, namely GIP (3–42) and GLP-1 (9–36) [5]. DPP-IV inhibitors are therefore useful for treating type 2 diabetes by reducing DPP-IV enzyme activity and increasing circulating concentrations of the active forms of both hormones. The advent of medical advances in treatment options has resulted in better glycaemic control. However, these options are often expensive and have secondary side effects that sometimes limit their use in wider and particularly poorer sections of society.

Herbal medicines are popular for a wide variety of ailments, and according to WHO, 75% of the global population uses herbs for basic healthcare needs [6]. Several medicinal plants and their formulations have gained attention in diabetes treatment [7]. An ethnobotanical and pharmacological survey provided useful information on plant species reported to possess antidiabetic activity that could be used as an adjunct treatment for T2DM therapy [8,9]. Folk medicines are popular and apparently effective for diabetes treatment in many regions of the world because of their availability, low cost and apparent safety and effectiveness [10]. However, few have been subjected to scientific scrutiny, and the mechanisms of action and nature of the active constituents are unknown. Several plants, including *Trigonella foenum greacum*, have been reported many times for their ability to treat T2DM [11,12]. Moreover, nearly 200 isolated compounds from different plant sources have been reported to lower blood glucose [9]. Some of these are alkaloids, carbohydrates, glycosides, flavonoids, steroids, terpenoids, peptides and amino acids, lipids, phenolic, glycopeptides and iridoids.

The gum of *Acacia arabica* is traditionally known as “Samghe arabi” in Persian medicine. The fruits have a long background of traditional use as an astringent, a diuretic, an antimicrobial and in wound healing therapy, as well as a liver tonic [13]. The gum is actively used as a dietary supplement for diabetes treatment in Ayurvedic medicine [14]. A previous study reported that fruits of *A. arabica* had no significant hypoglycaemic action in diabetic rabbits but lowered blood glucose in normal animals [15]. Several parts of *A. arabica* have been studied for hypoglycaemic effects [16,17,18]. A recent study also reported the potential effect of *A. arabica* on insulin resistance, blood glucose and lipid profile in streptozotocin (STZ)-induced diabetic rats [19]. None of these studies has provided a convincing and full account of the antidiabetic activity of *A. arabica*. Therefore, the present study was designed to fully investigate the pancreatic and extrapancreatic antidiabetic properties of *A. arabica* in vitro and in vivo to understand the mechanisms of action and nature of its bioactive compounds.

## 2. Results

### 2.1. Effects of Extract of A. arabica Bark on Insulin Release from BRIN-BD11 Cells

**Figure 1:**
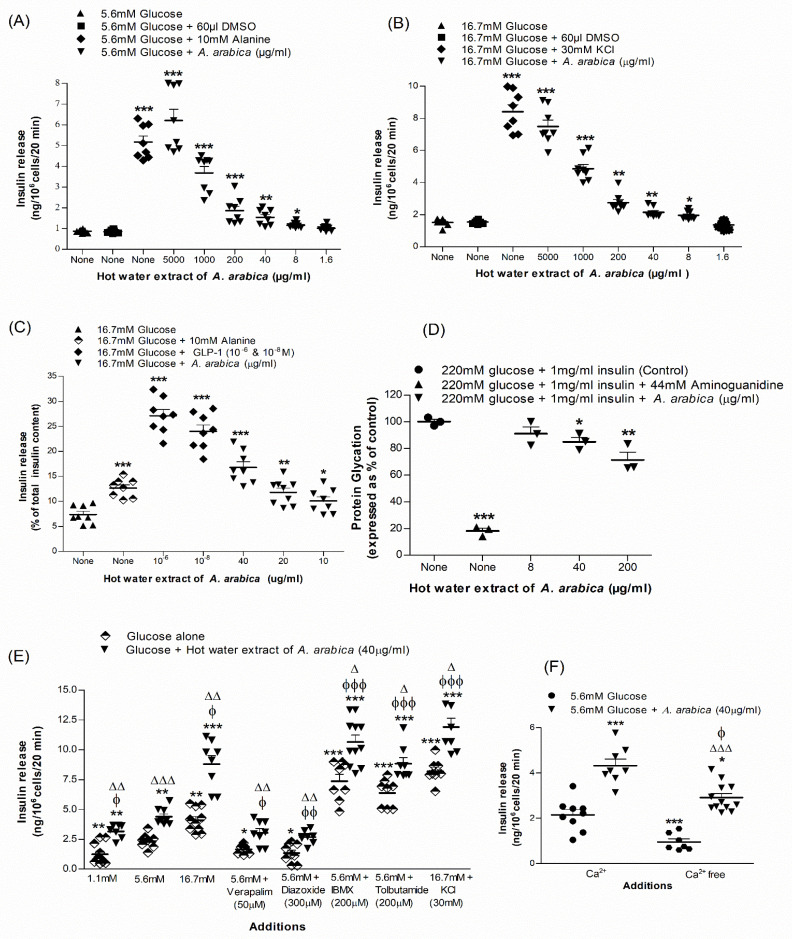
Dose-dependent effects of hot-water extract of *A. arabica* bark on insulin release from (**A**,**B**) BRIN-BD11 cells and (**C**) islets of Langerhans, (**D**) protein glycation, (**E**) insulin secretion in the presence of established stimulators or inhibitors and (**F**) absence of extracellular calcium. Values are the mean ± SEM for *n* = 4–8 for insulin release and *n* = 3 for protein glycation. * *p* < 0.05, ** *p* < 0.01 and *** *p* < 0.001 compared to control (5.6/16.7 mM glucose and 220 mM glucose + insulin (1 mg/mL)). ^ϕ^ *p* < 0.05, ^ϕϕ^ *p* < 0.01 and ^ϕϕϕ^ *p* < 0.001 compared to 5.6 mM glucose in the presence of the extract. ^Δ^ *p* < 0.05, ^ΔΔ^ *p* < 0.01 and ^ΔΔΔ^ *p* < 0.001 compared to respective incubation in the absence of the extract.

The basal rate of insulin release from BRIN-BD11 cells in the presence of 5.6 mM glucose was 0.87 ± 0.03 ng/10^6^ cells/20 min, and this rate increased to 4.45 ± 0.53 ng/10^6^ cells/20 min (*p* < 0.05; *n* = 8) in the presence of alanine (10 mM) (Figure 1A). At 16.7 mM glucose, basal insulin release was 1.50 ± 0.07 ng/106 cells/20 min, which was increased to 8.40 ± 0.44 ng/106 cells/20 min in the presence of 30 mM KCl (Figure 1B). Hot-water extract of *A. arabica* bark stimulated insulin release in a dose-dependent manner (1.6–5000 μg/mL) at 5.6 mM or 16.7 mM glucose (*p* < 0.05–0.001; Figure 1A,B). Extract at 5000 μg/mL produced maximum responses of 5.1- to 6.8-fold times the basal rate. However, the higher concentrations (200, 1000 and 5000 ug/mL) increased lactate dehydrogenase (LDH) release by 1.30–6.18-fold (Figure S1A,B). At extract concentrations of 1.6–40 μg/mL, no lactate dehydrogenase (LDH; cytosolic enzyme) release was observed, indicating the lack of deleterious effects on plasma membrane (Figure S1A,B).

### 2.2. Effects of Extract of A. arabica Bark on Insulin Release from Isolated Mouse Islets

Hot-water extract produced a substantial increase in insulin secretion from isolated mouse islets at 16.7 mM glucose (Figure 1C). A significant stimulation was produced at extract concentrations of ≥20 μg/mL (Figure 1C). The increase in insulin secretion induced by hot-water extract was moderately less than the positive control GLP-1 (10^−6^ and 10^−8^ M) in the presence of 16.7 mM glucose (Figure 1C).

### 2.3. Effects of Extract of A. arabica Bark on Glycation of Insulin

Hot-water extract evoked a 15–30% inhibition (*p* < 0.05–0.001; Figure 1D) of insulin glycation at 40–200 μg/mL. Aminoguanidine (44 mM) used as positive control inhibited glycation by 83% (*p* < 0.001; Figure 1D).

### 2.4. Insulinotropic Effects of Extract of A. arabica Bark in the Presence of Known Modulators of Insulin Release

*A. arabica* (40 µg/mL) extract was incubated with known modulators of insulin release to evaluate mechanisms responsible for the insulinotropic activity of the plant (Figure 1E). Insulin-releasing effects were partly reduced by the K^+^ channel activator, diazoxide (300 µM). Similar inhibition was observed in the presence of the L-type voltage-dependent Ca^2+^ channels blocker, verapamil (50 μM) (Figure 1E). The insulin-releasing action was preserved in incubations with tolbutamide and a 30 mM depolarising concentration of KCl (Figure 1E). The phosphodiesterase inhibitor, isobutylmethylxanthine (IBMX), also enhanced insulin-releasing activity (*p* < 0.001; Figure 1E). Dependency of the plant’s insulinotropic effect on [Ca^2+^]i was confirmed by incubations in the absence of Ca^2+^, which diminished insulin release by 32% (Figure 1F).

### 2.5. Effects of Extract of A. arabica Bark on Membrane Depolarisation and Intracellular Calcium Concentration in BRIN-BD11 Cells

**Figure 2:**
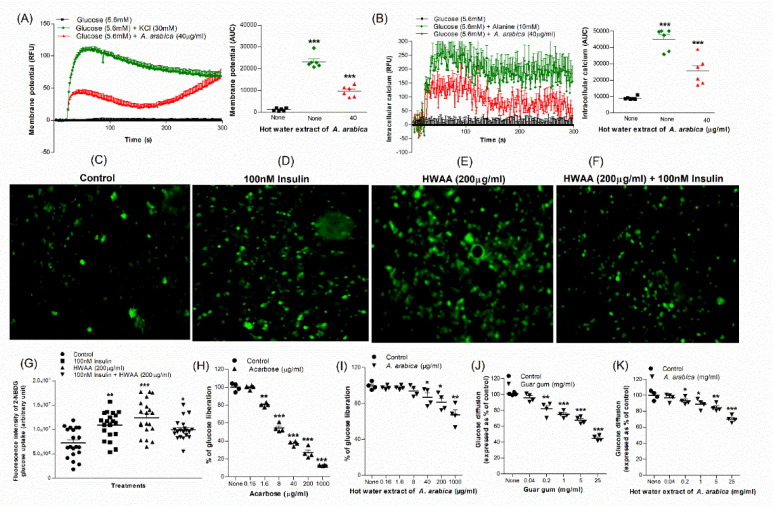
Effects of hot-water extract of *A. arabica* bark on (**A**) membrane potential and (**B**) intracellular calcium in BRIN BD11 cells, (**C**–**G**) glucose uptake in differentiated 3T3L1 adipocyte cells, (**H**) acarbose, (**I**) starch digestion, (**J**) guar gum and (**K**) glucose diffusion in vitro. Changes in fluorescence intensity in differentiated 3T3L1 adipocytes incubated with extract in the (**E**) absence or (**F**) presence of 100 nM insulin. Images were taken at 10× magnification. Values are the mean ± SEM for *n* = 6 for membrane potential and intracellular calcium and *n* = 4 (~20 values of fluorescence intensity per group for glucose uptake) for glucose uptake, starch digestion and glucose diffusion. * *p* < 0.05, ** *p* < 0.01 and *** *p* < 0.001 compared to control.

KCl (30 mM) and alanine (10 mM) were used as positive controls that elicited significant depolarisation of membrane potential and elevation of [Ca^2+^]i concentration, respectively (*p* < 0.001; Figure 2A,B). Hot-water extract also evoked membrane depolarisation and an increase in [Ca^2+^]i concentration (*p* < 0.001; Figure 2A,B).

### 2.6. Effects of Extract of A. arabica Bark on Glucose Uptake and Insulin Action

Glucose uptake and insulin action were studied using 3T3L1 differentiated adipocyte cells and a fluorescent glucose analogue (Figure 2C–G). In the microscopic fluorescence analysis, *A. arabica* extract enhanced glucose uptake significantly compared to control (*p* < 0.05–0.001; Figure 2G). The effect was not potentiated by 100 nM insulin. Insulin alone stimulated glucose uptake by 1.5-fold (*p* < 0.01; Figure 1G) compared to control.

### 2.7. Effects of Extract of A. arabica Bark on Starch Digestion

Acarbose (1 mg/mL) used as positive control inhibited enzymatic glucose liberation from starch by 87% (Figure 2H). The hot-water extract significantly inhibited starch digestion at concentrations of 40–1000 µg/mL, with a maximum of 32% inhibition (*p* < 0.01) at 1000 µg/mL (Figure 2I).

### 2.8. Effects of Extract of A. arabica Bark on Glucose Diffusion In Vitro

Hot-water extract of *A. arabica* (mg/mL) had significant inhibitory effects on glucose diffusion after 24 h of incubation (Figure 2K). The maximal inhibition of 30% was observed at 25 mg/mL (*p* < 0.05–0.01; Figure 2K). Guar gum (25 mg/mL) used as positive control inhibited glucose movement by a maximum of 55% (Figure 2J).

### 2.9. Effects of Extract of A. arabica Bark on DPP-IV Enzyme Activity In Vitro

**Figure 3:**
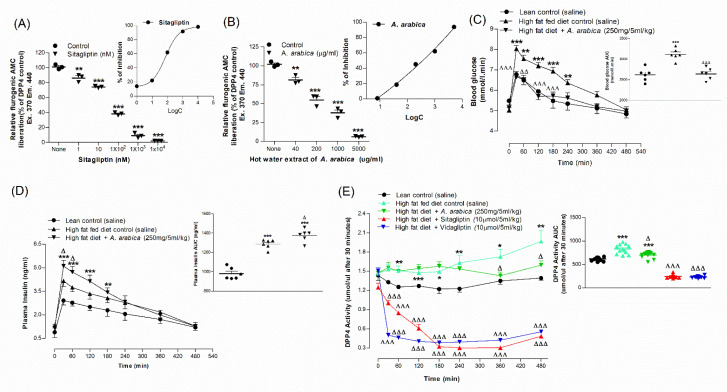
Acute effects of (**A**) sitagliptin and (**B**) hot-water extract of *A. arabica* bark on DPP-IV enzyme activity in vitro, (**C**) glucose tolerance, (**D**) plasma insulin and (**E**) plasma DPP-IV in high-fat-fed rats. Parameters were measured prior to and after oral administration of glucose alone (18 mmol/kg body weight, control) or with simultaneous *A. arabica* extract (250 mg/5 mL/kg body weight). Established DPP-IV inhibitors, sitagliptin and vildagliptin, were used as positive controls. Values are the mean ± SEM, *n* = 3 for DPP-IV enzyme activity in vitro and *n* = 6 for glucose tolerance, plasma insulin and DPP-IV in vivo. * *p* < 0.05, ** *p* < 0.01 and *** *p* < 0.001, compared to normal control and ^Δ^ *p* < 0.05, ^ΔΔ^ *p* < 0.01 and ^ΔΔΔ^ *p* < 0.001 compared to high-fat-fed control.

Sitagliptin, an established drug (10 µM), inhibited the enzymatic AMC liberation from the DPP-IV substrate, Gly-Pro-AMC, by 98% (Figure 3A). Hot-water extract significantly inhibited the DPP-IV enzyme by 18–93% (*p* < 0.01–0.001, Figure 3B) at 40–5000 µg/mL.

### 2.10. Acute Effects of Hot-Water Extract of A. arabica Bark on Oral Glucose Tolerance and Plasma DPP-IV in High-Fat-Fed Rats

A single dose of *A. arabica* hot-water extract (250 mg/5 mL/kg; body weight) elicited a significant (*p* < 0.05–0.001) decrease in blood glucose at 30, 60, 120 and 180 min compared to control rats (Figure 3C). The extract also significantly increased plasma insulin at 30 and 60 min (*p* < 0.05; Figure 3D). AUC analysis revealed a 13% decrease (*p* < 0.001; Figure 3C) in blood glucose excursion and a 10% increase (*p* < 0.05; Figure 3D) in plasma insulin. The extract also inhibited plasma DPP-IV enzyme activity (*p* < 0.05) in a time-dependent manner (Figure 3E). AUC calculations also revealed an 11% decrease in DPP-IV enzyme activity (*p* < 0.05; Figure 3E). Sitagliptin and vildagliptin (10 μmol/5 mL/kg), used as gold-standard drugs, produced 70–75% reductions in plasma DPP-IV enzyme activity (*p* < 0.001; Figure 3E).

### 2.11. Effects of Twice-Daily Oral Administration of Hot-Water Extract of A. arabica Bark on Body Weight and Metabolism in High-Fat-Fed Rats

**Figure 4:**
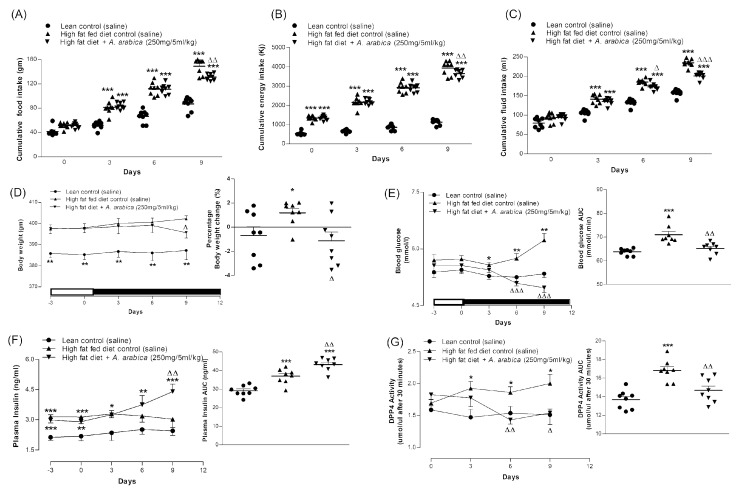
Effects of 9-day, twice-daily oral administration of hot-water extract of *A. arabica* bark on (**A**) food intake, (**B**) energy intake, (**C**) fluid intake, (**D**) body weight, (**E**) blood glucose, (**F**) plasma insulin and (**G**) DPP-IV enzyme activity in high-fat-fed rats. Parameters were measured prior to and after oral administration of *A. arabica* bark (250 mg/5 mL/kg, body weight) twice daily. Values are the mean ± SEM for *n* = 8 rats. * *p* < 0.05, ** *p* < 0.01 and *** *p* < 0.001 compared to lean control. ^Δ^ *p* < 0.05, ^ΔΔ^ *p* < 0.01 and ^ΔΔΔ^ *p* < 0.001 compared to high-fat-fed control at corresponding time points.

**Figure 5:**
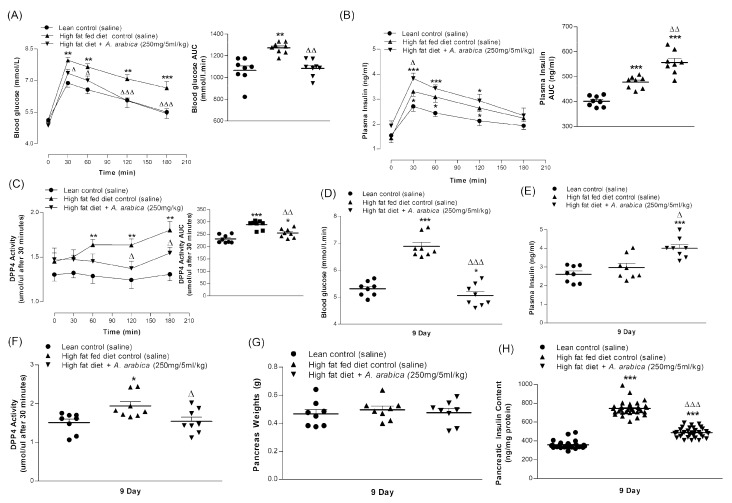
Chronic effects of twice-daily oral administration of hot-water extract of *A. arabica* bark on (**A**) glucose tolerance, (**B**) plasma insulin and (**C**) plasma DPP-IV on Day 6 and (**D**) blood glucose, (**E**) plasma insulin, (**F**) plasma DPP-IV, (**G**) pancreas weight and (**H**) pancreatic insulin content on Day 9 in high-fat-fed rats. Parameters were measured after treatment for 6 or 9 days with twice-daily oral administration of hot-water extract of *A. arabica* bark (250 mg/5 mL/kg body weight). Values are the mean ± SEM with *n* = 8 (~32 per group for pancreatic insulin content). * *p* < 0.05, ** *p* < 0.01 and *** *p* < 0.001 compared to lean control. ^Δ^ *p* < 0.05, ^ΔΔ^ *p* < 0.01 and ^ΔΔΔ^ *p* < 0.001 compared to high-fat-fed control at corresponding time points.

The treatment for 9 days with *A. arabica* extract (250 mg/5 mL/kg; b.w.) resulted in significant improvements in all parameters measured (Figure 4A–C,E,F and Figure 5D,E). Body weight and cumulative food intake were decreased by 9–12% (*p* < 0.05–0.01; Figure 4A,D). The extract also reduced fluid intake and blood glucose by 9–14% (*p* < 0.05–0.001; Figure 4C,E and Figure 5D), with a clear-cut effect from 6 days onwards. The extract also increased plasma insulin by 14% (*p* < 0.01; Figure 4F and Figure 5E) and inhibited DPP-IV enzyme activity from Day 6 onwards (*p* < 0.01, Figure 4G and Figure 5F).

### 2.12. Effects of Twice-Daily Oral Administration of Hot-Water Extract of A. arabica Bark on Glucose Tolerance in High-Fat-Fed Rats

After 6 days of treatment with *A. arabica* extract (250 mg/5 mL/kg; b.w.), oral glucose tolerance was significantly (*p* < 0.05–0.01) improved from 30 min onward (Figure 5A). The effect was also associated with an increase in plasma insulin after 30 min (*p* < 0.05; Figure 5B). AUC analysis showed a 16% reduction in blood glucose (*p* < 0.01; Figure 5A) and a 15% increase in insulin responses (*p* < 0.01; Figure 5B) compared to the high-fat-fed control rats. The extract also inhibited plasma DPP-IV from 60 min onwards (Figure 5C). AUC data showed a 11% decrease (*p* < 0.01) in enzyme activity compared to high-fat-fed control rats (Figure 5C).

### 2.13. Effects of Twice-Daily Oral Administration of Hot-Water Extract of A. arabica Bark on Pancreatic Insulin Content in High-Fat-Fed Rats

No significant changes were observed in the pancreas weight of treated or untreated high-fat-fed compared with lean control rats (Figure 5G). Pancreatic insulin content in high-fat-fed rats was increased by 54% (*p* < 0.001) (Figure 5H). *A. arabica* decreased pancreatic insulin compared to high-fat-fed controls (*p* < 0.001), but it was still increased by 35% (*p* < 0.001) compared to lean rats (Figure 5H).

### 2.14. Effects of Twice-Daily Oral Administration of Hot-Water Extract of A. arabica Bark on Islet Morphology in High-Fat-Fed Rats

**Figure 6:**
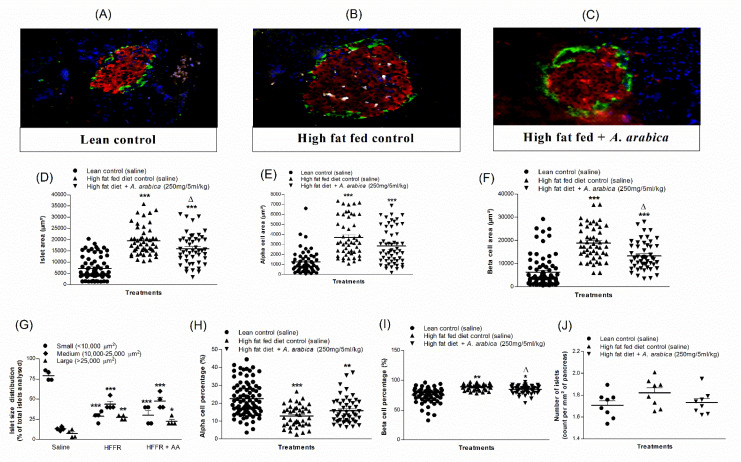
Effects of 9-day twice-daily oral administration of hot-water extract of *A. arabica* bark on islet morphology in high-fat-fed rats. Representative images of (**A**) lean control, (**B**) high-fat-fed control and (**C**) high-fat-fed plus hot-water extract of *A. arabica* (250 mg/5 mL/kg) in rats showing insulin in red, glucagon in green and DAPI in blue, (**D**) islet area, (**E**) alpha-cell area, (**F**) beta-cell area, (**G**) islet size distribution, (**H**) alpha-cell percentage, (**I**) beta-cell percentage and (**J**) number of islets (per mm^2^), respectively. Values are the mean ± SEM for *n* = 8 (~50 islets per group). * *p* < 0.05, ** *p* < 0.01 and *** *p* < 0.001 compared to lean control. ^Δ^ *p* < 0.05 compared to high fat fed alone (control).

Representative images of islets of normal, high-fat-fed control and treated high-fat-fed rats are shown in Figure 6A–C). High-fat feeding did not change the number of islets per mm^2^ in the pancreas (Figure 6J), but a significant increase in islet area was observed compared to the normal rats (*p* < 0.001; Figure 6D). High-fat-fed rats also exhibited a significant (*p* < 0.001) increase in alpha-cell and beta-cell areas (Figure 6E,F). Treatment with *A. arabica* extract significantly reduced overall islet area and beta-cell area (*p* < 0.05; Figure 6D,F). As shown in Figure 6G, high-fat-fed rats possessed a greater number of large- and medium-sized islets cells compared to normal control rats. High-fat-fed groups had a higher percentage of β cells and a lower proportion of α cells compared with normal rats (*p* < 0.05–0.01; Figure 6H,I).

### 2.15. Acute Effects of Peak Samples of A. arabica Bark on Insulin Release from BRIN-BD11 Cells

**Figure 7:**
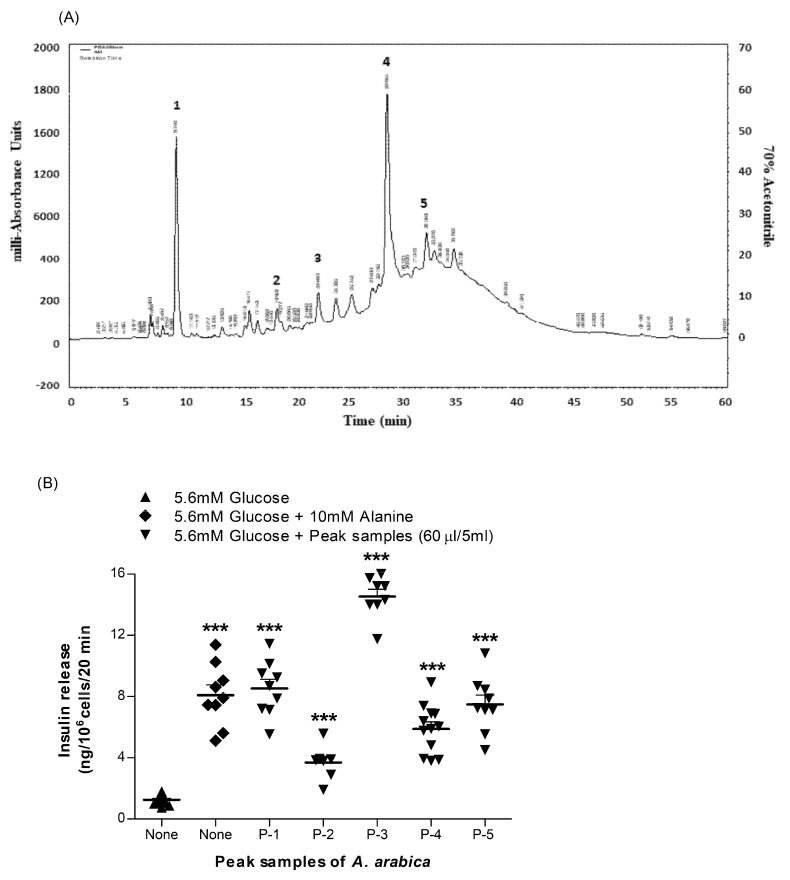
Representative (**A**) HPLC profile and (**B**) insulin-releasing effects in BRIN BD11 cells of peak samples (1–5) of hot-water extract of *A. arabica* bark. The crude extract was chromatographed at a flow rate of 1.0 mL/min on a (10 × 250 mm) semipreparative 5 μm C-18 column (Phenomenex, UK). The concentration of the eluting solvent was raised using linear gradients of acetonitrile (0–20% from 0 to 10 min, 20–70% from 10 to 40 min and 70–20% from 40 to 60 min). Compounds were detected by measurement of absorbance at 254 nm, and major peaks labelled P1-P5 were assessed for insulin-releasing activity. Values are the mean ± SEM for *n* = 8. *** *p* < 0.001 compared to control.

The five major and clearly defined peak fractions of *A. arabica* bark extract from RP-HPLC (Figure 7A) were assayed for insulin-secretory activity using BRIN-BD11 cells as described above. As shown in Figure 7B, all five peak fractions (P-1, P-2, P-3, P-4 and P-5) significantly stimulated insulin release (*p* < 0.001), as did the 10 mM alanine-positive control. Only P-3 was associated with cytotoxicity, as evidenced by the 2.1-fold increase in cellular LDH release (Figure S1C).

### 2.16. Purification and Structural Characterisation of Purified Extract of A. arabica Bark

Compounds were isolated using RP-HPLC and partially characterised by LC-MS (Figure 7A and Table 1). The RP-HPLC and LC-MS analyses predicted that Peaks 1, 2 and 3 were quercetin, catechin and kaempferol, respectively. In the case of quercetin, compound identity was further probed by NMR. Isolated compound quercetin was analysed through ^1^H and ^13^C NMR for characterisation: C_15_H_10_O_7_ was obtained as a yellow powder, λmax = 360, 256 nm; EI-MS *m/z* 301.2 Da [M]; 600 MHz, CD3OD, ^1^H-NMR (δ in ppm); 6.19 (d, ^1^H, *J* = 2 Hz, H-6), 6.40 (d, ^1^H, *J* = 2 Hz, H-8), 6.88 (d, ^1^H, *J* = 8.4 Hz, H-5′), 7.64 (d, ^1^H, *J* = 7.4 Hz, H-6′) and 7.80 (d, ^1^H, *J* = 2 H, H-2′). ^13^C NMR (600 MHz, CD_3_OD, δ in ppm); 147.4 (C-2), 135.8 (C-3), 175.9 (C-4), 161.1 (C-5), 97.8 (C-6), 164.1 (C-7), 93.0 (C-8), 156.8 (C-9), 103.1 (C-10), 122.3 (C-1′), 115.6 (C-2′), 145.6 (C-3′), 147.7 (C-4′), 116.1 (C-5′) and 119.2 (C-6′).

**Figure 8:**
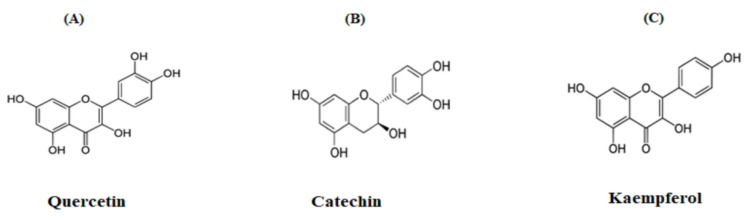
Isolated compounds (**A**) quercetin, (**B**) catechin and (**C**) kaempferol of Peak-1, -2 and -3 samples obtained from RP-HPLC of hot-water extract of *A. arabica* bark via LC-MS analysis. Proton-decoupled natural abundance ^1^H-NMR and C^13^-NMR of the Peak-1 sample of *A. arabica* bark (obtained from a chromatograph over the period of 70% acetonitrile from 10 to 40 min with a retention time of 9.7 min) at 40 °C. The spectrum values were obtained at 600 MHz after 119,044 transients (14 h) by the pulsed Fourier-transform method on a Varian XL-100 A spectrometer. The representative structures of flavonoids, corresponding to the molecular formula of quercetin, catechin and kaempferol, are C_15_H_10_O_7,_ C_15_H_14_O_6_ and C_15_H_10_O_6_.

The ^1^H-NMR spectrum of the isolated compound showed aromatic hydrogen groups from 6.19 to 7.80 ppm. The ^13^C-NMR spectrum showed a carbonyl group at 175.9 ppm and an aromatic carbon group from 93.0 to 164.1 ppm. The structure was verified via comparison with evidence from the literature [20,21]. Molecular structures of tentatively identified compounds are outlined in Figure 8A–C.

### 2.17. Acute Effects of Isolated Compounds Quercetin and Kaempferol on Insulin Release from BRIN-BD11 Cells

**Figure 9:**
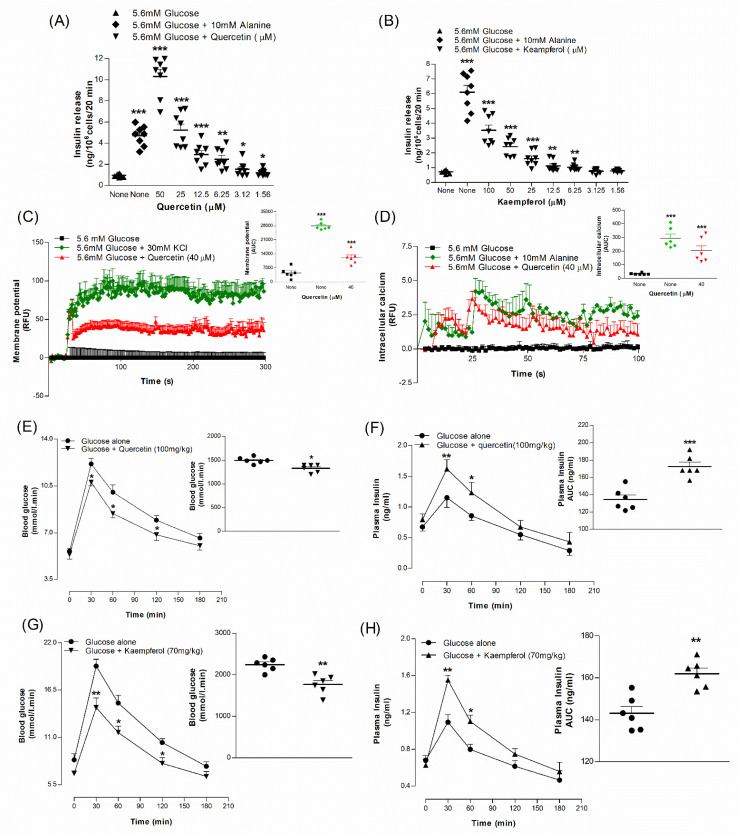
Insulin-releasing effects of (**A**) Quercetin and (**B**) Kaempferol, (**C**) membrane potential and (**D**) intracellular calcium from BRIN-BD11 cells, (**E**,**G**) glucose tolerance and (**F**,**H**) plasma insulin. Mice were fasted for 12 h and administered glucose (18 mmol/kg body weight) by oral gavage with or without (**E**,**F**) simultaneous quercetin (100 mg/kg b.w.) and (**G**,**H**) kaempferol (70 mg/kg b.w.). Values are the mean ± SEM for *n* = 8 for insulin release, *n* = 6 for membrane potential, intracellular calcium, glucose tolerance and plasma insulin. * *p* < 0.05, ** *p* < 0.01 and *** *p* < 0.001 compared to control.

Quercetin and kaempferol isolated from *A. arabica* bark were tested to confirm insulin-secretory activity using BRIN-BD11 cells (Figure 9A,B). Alanine (10 mM) was again used as positive control. Quercetin stimulated insulin secretion at 1.56–50 µM (*p* < 0.05–0.001, Figure 9A), but at 50 µM increased LDH release by 2.1-fold (Figure S1D). Kaempferol also increased insulin release in a dose-dependent manner (6.25–100 µM; *p* < 0.01–0.001, Figure 9B) from BRIN-BD11 cells. At concentrations ≥100 µM, this was associated with cytotoxicity and increased LDH release by 1.25-fold (Figure S1E).

### 2.18. Effects of Isolated Compound Quercetin on Membrane Depolarisation and ([Ca^2+^]_i_ in BRIN-BD11 Cells

Quercetin (40 μM) depolarised BRIN-BD11 cells in the presence of 5.6 mM glucose (Figure 9C). Similarly, quercetin at 40 μM substantially (*p* < 0.001) increased [Ca^2+^]_i_ compared to control (Figure 9D). KCl (30 mM) and alanine (10 mM) were used as positive controls.

### 2.19. Acute Effects of Synthetic Compound Quercetin and Kaempferol on Oral Glucose Tolerance in Mice

Oral glucose tolerance was significantly improved (*p* < 0.05–0.01) at 30, 60 and 120 min when quercetin (100 mg/kg) or kaempferol (70 mg/kg) were co-administered with glucose (18 mmol/kg body weight) to mice (Figure 9E,G). Plasma insulin concentrations were also increased at 30 and 60 min (*p* < 0.05–0.01; Figure 9F,H). AUC analysis showed an 11–22% (*p* < 0.01) decrease in blood glucose and a 22–24% (*p* < 0.01) increase in plasma insulin responses, respectively (Figure 9E–H).

## 3. Discussion

*A. arabica*, commonly known as babul, has been reported to possess antidiabetic properties by traditional healers and recent scientific reports [22]. However, the validity of these claims and the molecular mechanisms underpinning its antidiabetic activity have not been elucidated [23,24]. To address this, we utilised a platform of in vitro tests and high-fat-fed rats to test potential antihyperglycaemic actions of hot-water extract of *A. arabica* bark.

We first evaluated the insulinotropic activity of *A. arabica* using clonal rat BRIN-BD11 cells and isolated mouse islets. This revealed stimulatory concentration-dependent insulin-secretory effects at nontoxic concentrations. Further assessment was made using fluorescent indicator dyes and known modulators of β-cell function, including diazoxide, verapamil, Ca^2+^ depletion, tolbutamide, KCl and IBMX. These studies revealed that the action of *A. arabica* involved the closure of K-ATP channels, membrane depolarisation, the opening of voltage-dependent calcium channels, the influx of Ca^2+^ and the elevation of intracellular Ca^2+^. Stimulatory effects nevertheless persisted in β cells depolarised by tolbutamide or 30 mM KCl, suggesting additional actions such as the activation of adenylate cyclase, which is supported by positive potentiation by the phosphodiesterase inhibitor, IBMX [25].

Insulin works primarily on skeletal muscle and adipose tissue for postprandial glucose regulation [26]. GLUT4 translocation is reduced, and an inadequate or defective signal leads to the development of insulin resistance [27]. Agents that can resolve insulin resistance are therefore of great utility for the treatment of T2DM [28]. In this study, *A. arabica* increased the glucose uptake by 3T3L1 adipocytes. The mechanisms responsible for such action need further clarification but might involve the activation of Akt and p70 kinase, as this has been shown to increase glycogen, lipid, and protein synthesis and in turn promote glucose uptake [29].

Several factors are involved in the pathophysiology of diabetes and its complications, such as the hyperglycaemic-induced glycation of structural and functional proteins [30]. Previous in vivo studies have reported that insulin can be glycated and reduces its biological activity by approximately 10%, thereby contributing to insulin resistance [31,32]. Inhibition of the glycation of insulin and other proteins is therefore a desirable feature of any antidiabetic remedy. In this study, *A. arabica* decreased insulin glycation in vitro in a concentration-dependent manner. This might reflect the antioxidant properties and phytoconstituents of the plant such as vitamin C, flavonoids, glycosides, quercetin and gallic acids [33,34].

*A. arabica* was tested for its effects on the in vitro enzymatic digestion of starch by α-amylase and α-glucosidase, leading to the liberation of glucose. Acarbose, an established α-glucosidase inhibitor used as a reference standard, inhibited glucose liberation significantly in a concentration-dependent manner. *A. arabica* also caused significant concentration-dependent inhibition of glucose liberation from starch. Previous studies have reported that flavonoids are very effective in reducing the α-amylase activity and slowing down starch digestion [35]. It has also been reported that *A. arabica* contains a high fibre content [36], which may slow gastric emptying.

A decrease in the absorption and diffusion of glucose from the gastrointestinal tract is one of the many reasons for plants to exhibit antihyperglycaemic activity [37]. We used a dialysis-based method to study the effects of plant extract on the diffusion of glucose through an artificial barrier. Although this method has certain limitations, such as being unphysiological and needing lengthy dialysis time (22–24 h), this technique is a simple and effective means to study the effects of viscosity on glucose diffusion. The glucose absorption blocker, guar gum, was used as positive control. In this system, *A. arabica* extract elicited significant concentration-dependent inhibition of glucose movement through the dialysis membrane.

High-fat diet-induced obese diabetic rodents are frequently used as models for investigating both the acute and chronic effects of plant extract and pharmaceutical products [38]. Oral administration of *A. arabica*, together with glucose to high-fat-fed rats, improved glucose tolerance, decreased circulating DPP-IV and augmented the accompanying plasma insulin response. In the follow-up chronic 9-day study, *A. arabica* elicited significant improvements in food intake, body weight, non-fasting glucose, glucose tolerance, plasma insulin and circulating DPP-IV. These observations support earlier studies showing that *A. arabica* decreased hyperglycaemia, TC, TG, LDL-C and MDA and increased HDL-C and Co-Q10 in STZ-induced diabetic rats [19].

The observation that benefits of *A. arabica* may extend to STZ rats typified by beta-cell destruction suggests a possible positive effect on islet morphology and beta-cell mass. Because we showed that the plant inhibits DPP-IV both in vitro and in vivo, the incretin hormones GLP-1 and GIP may play a role in mediating such effects, as both peptides are known to exert positive effects on β-cell proliferation, apoptosis, and islet cell transdifferentiation [39,40,41]. Thus, by inhibiting DPP-IV, *A. arabica* will block the degradation of GIP and GLP-1 to their inactive metabolites GLP-1 (9–36) and GIP (3–42) [42,43], thereby promoting concentrations of their active forms. Several studies claim that phytochemicals in many plant species have the potential to inhibit DPP-IV by directly blocking the DPP-IV enzyme ligand-binding site [44,45]. Based on our phytochemical analysis of *A. arabica* extract, it seems likely that flavonoids are the active compounds responsible for DPP-IV enzyme inhibitory action. However, further studies are clearly needed to confirm this hypothesis.

Interestingly, histological analysis of the pancreas of high-fat-fed rats in the present study revealed that *A. arabica* countered the diet-induced increases in islet, beta-cell and alpha-cell areas associated with insulin resistance. The pancreatic insulin content was also increased in the high-fat-fed group, being associated with a greater number of large- and medium-sized islets with no overall change in the total number of islets per mm^2^. It seems likely that these effects of *A. arabica* are not directly mediated by plant phytochemicals but are a consequence of the amelioration of hyperglycaemia and improvement of insulin resistance. However, further studies would be useful to confirm this.

Due to increasing interest in the availability of biologically or pharmacologically active compounds, the search for phytochemicals responsible for the bioactivity of crude plant extract has gained significant prominence. In this study, *A. arabica* crude extract was further analysed to isolate, identify, and characterise molecular compounds with insulinotropic and antidiabetic activity. Five major peaks (P-1 to P-5) were isolated by RP-HPLC and shown to stimulate insulin release from clonal β cells. Further analysis showed identity similar to the known phytochemicals quercetin (P-1), catechin (P-2) and kaempferol (P-3) [46]. The isolated fraction P-1 (quercetin) was a yellow amorphous powder that was further characterised by NMR. This revealed that the ^1^H-NMR spectrum of the isolated compound had aromatic hydrogen groups from 6.19–7.80 ppm. The ^1^H-NMR spectrum showed two peaks at 6.19 (d, ^1^H, *J* = 2 Hz) and 6.40 ppm (d, ^1^H, *J* = 2 Hz), consistent with the meta protons on the A ring and at 6.88 (d, ^1^H, *J* = 8.4 Hz), 7.64 (d, ^1^H, *J* = 7.4 Hz) and 7.80 (d, ^1^H, *J* = 2 Hz) corresponding to the catechol protons on the B ring. The ^13^C-NMR spectrum showed a carbonyl group at 175.9 ppm and an aromatic carbon group from 93.0 to 164.1 ppm. These data are consistent with those reported in the literature for the compound quercetin [20,21]. Quercetin and kaempferol significantly increased insulin release in a concentration-dependent manner and improved both glucose tolerance and plasma insulin responses in mice. Quercetin (P-1), catechin (P-2) and kaempferol (P-3) have been suggested to have significant antidiabetic potential [47], and the presence of these phytochemicals might be responsible for the substantial antidiabetic activity of *A. arabica* bark.

## 4. Materials and Methods

### 4.1. Collection and Preparation of Plant Extracts

The bark of *A. arabica* was collected from Jahangirnagar University, Dhaka, Bangladesh, and Botanical Accession Number 43,756 was assigned by the National Herbarium. Barks were processed for hot-water extraction, as described previously [48]. The final extracted semisolid, sticky residue of *A. arabica* bark was freeze-dried using a freeze dryer (Varian 801 LY-3-TT, Varian, Lexington, MA, USA) and stored at 4 °C until use. Flavonoids, including quercetin, catechin and kaempferol, were obtained from Sigma-Aldrich (Poole, UK).

### 4.2. In Vitro Insulin-Releasing Studies

Insulin-releasing BRIN BD11 cells were generated by the electrofusion of New England Deaconess Hospital rat pancreatic beta cells with immortal clonal RINm5F islet cells [49]. The insulin secretion from clonal BRIN-BD11 cells and mouse islets was observed as per a previous description [50]. Plant extract was incubated with or without known modulators of insulin secretion at 1.1, 5.6 or 16.7 mM glucose, respectively. Dextran-coated charcoal radioimmunoassay was used to measure insulin in the aliquoted supernatant samples, which were stored at −20 °C [48]. For the analysis of insulin content, islets were extracted at 4 °C for 24 h, and supernatant samples collected after centrifugation for 2 min at 1200 rpm were stored at −20 °C prior to radioimmunoassay [49].

### 4.3. Membrane Potential and Intracellular Calcium ([Ca^2+^]i)

To measure the intensity of membrane depolarisation and [Ca^2+^]i of BRIN-BD11 cells with *A. arabica* extract, we used the FLIPR Membrane Potential and [Ca^2+^]i Assay Kit (Molecular Devices, Sunnyvale, CA, USA) [49]. In brief, clonal pancreatic beta cells were seeded on 96-well plates for 18 h at 37 °C to allow attachment. The medium was then removed, and 100 μL KRBB of 5.6 mM glucose at 37 °C was added. After 10 min, 100 μL of FLIPR membrane potential or calcium dye was added, and the cells were incubated for 60 min at 37 °C. Signal intensity changes were measured using FlexStation 3 (Molecular Devices, Sunnyvale, CA, USA).

### 4.4. Cellular Glucose Uptake

The 3T3 L1 differentiated adipose cells were used to estimate the glucose uptake [51]. The cells were seeded in 24-well plates and kept in an incubator at 37 °C for 30 min with *A. arabica* in the presence or absence of 100 nM insulin before incubation with 2-NBDG (50 nM) for 5 min. Every single well was washed twice with ice-cold PBS, and slides were covered with three to four coverslips. By using a microscope with 10× magnification, four images of the coverslips were taken in order to measure glucose uptake with fluorescence intensity.

### 4.5. Insulin Glycation

Insulin glycation in the presence of *A. arabica* was assessed following a previous description [52]. During the experiment, incubation of D-glucose (246.5 mM) with human insulin (1 mg/mL) and NaBH3CN (0.0853 gm/mL) with or without HWAA (8, 40, 200 μg/mL) was performed. A day later, 0.5 M acetic acid was added to stop the reaction. RP-HPLC was used to measure glycated and nonglycated insulin [31].

### 4.6. DPP-IV Enzyme Activity In Vitro

The DPP-IV enzyme inhibitory actions of *A. arabica* were estimated following an earlier report [43]. The chemical reagents, including 8 mU/mL of DPP-IV enzyme and 200 μM of substrate (Gly-Pro-AMC), were incubated with treatment groups in 96-well plates and processed as per a previous description [41]. Visual changes in the fluorescence were analysed by excitation at 370 nm and emission at 440 nm with a 2.5 nm slit width by FlexStation 3 (Molecular Devices, Sunnyvale, CA, USA).

### 4.7. Starch Digestion

Extract effects on starch digestion [53] were measured by incubating *A. arabica* or acarbose in starch solution (100 mg) (Sigma-Aldrich, St. Louis, MO, USA). Heat-stable α-amylase (0.01%) from *Bacillus leicheniformis* and amyloglucosidase (0.1%) from Rhizopus mould (Sigma-Aldrich, St. Louis, USA) were added to the mixture after dilution. Final samples were stored for further analysis of glucose release by utilising the GOD/PAP method (Randox GL 2623).

### 4.8. Glucose Diffusion

Cellulose ester (CE) dialysis tubes (20 cm × 7.5 mm, MWCO: 2000, Spectrum, The Netherland) were employed to estimate glucose diffusion in vitro following a recent study [54]. The solution of 220 mM glucose (2 mL) was loaded into the tubes in the presence or absence of treatments (including *A. arabica* and guar gum). The tubes with closed ends were placed inside 0.9% NaCl (45 mL) solution with continuous shaking for 24 h at 37 °C. The amount of glucose that pierced the tube was assessed.

### 4.9. Animals

A high-fat diet (20% protein, 45% fat and 35% carbohydrate, 26.15 KJ/g total energy percentage, Special Diet Service, Essex, UK) was fed to Sprague–Dawley male rats for 150–180 days prior to the study. A standard diet (10% fat, 30% protein and 60% carbohydrate, 12.99 KJ/g total energy, Trouw Nutrition, Cheshire, UK) was fed to lean control rats. Additional experiments to test the effects of quercetin and kaempferol were conducted to see the improvement in glucose tolerance in 6-week old normal male Swiss albino mice (Envigo).

### 4.10. Ethical Approval

The Animal Welfare and Ethical Review Board (AWERB) at Ulster University approved the studies, performed based on the UK Animals (Scientific Procedures) Act 1986 and EU Directive 2010/63EU. Precautions were taken to avoid any discomfort with rodents.

### 4.11. Oral Glucose Tolerance and Plasma DPP-IV

Fasted high-fat-fed rats (12 h) or mice (6 h) were used to see the impact of HWAA (250 mg/5 mL/kg) and isolated compounds (quercetin and kaempferol) (100 and 70 mg/kg) on blood glucose control [49]. An oral glucose tolerance test after the administration of glucose with or without simultaneous treatment with *A. arabica* was performed. Blood collected from tail tips at different time points mentioned in the figures was used for glucose (Figure 3 and Figure 5 for 6 h and Figure 9 for 12 h) and plasma insulin measurement [55]. Enzymatic (DPP-IV) assay in plasma was performed following a previous report [43], as given above.

### 4.12. Glucose Homeostasis in Obese Rats

*A. arabica* (250 mg/5 mL/kg body weight) or 0.9% (*w*/*v*) saline vehicle was administered orally twice a day in high-fat-fed rats for 9 successive days. At a particular time interval, parameters such as blood glucose and plasma insulin were assessed. Following 6 days of treatment, OGTT (18 mmol/kg) was performed in 12 fasted rats. At the completion of the study, pancreatic tissues were extracted according to a previous report [49].

### 4.13. Islet Morphology Studies in Obese Rats

Pancreatic tissues were sliced into 5 to 8 μM sections, fixed, processed, stained and analysed [56]. Sections were incubated with both a primary antibody (mouse anti-insulin (1:500) and guinea pig anti-glucagon (1:400)) and secondary antibody mixture (Alexa Fluor 594 goat anti-mouse antibody and Alexa Fluor 488 goat anti-guinea pig antibody) at 4 °C (overnight) and room temperature, respectively. Subsequent to staining the nucleus with 4′,6-diamidino-2-phenylindole (DAPI), the slides were mounted and analysed following a previous description [2].

### 4.14. Purification of Crude Extracts

The filtered extract was injected into a Vydac 218TP1022 (Grace, Deerfield, IL, USA) preparative stainless-steel column (C-18, 10 μm) (22 × 250 mm^2^) at a 5.0 mL/min flow rate with 0.12% (*v*/*v*) TFA/water. Acetonitrile concentration in the eluting solvent was mounted using linear gradients to 20% for 10 min and to 70% over a period of 40 min. Major peaks were tested for insulinotropic activity, as detailed above, and positive fractions were purified again at a flow rate of 1.0 mL/min following recent studies [2,57].

### 4.15. Determination of Molecular Weight

LC-MS was implemented to measure the molecular weight of selected peak fractions of *A. arabica* obtained from RP-HPLC via ESI-MS. A Spectra System LC (Thermo Separation Products) containing a Kinetex 5 µm F5 LC column (150 × 4.6 mm^2^) was used to identify the identity of the peak fraction as previously reported [58].

### 4.16. Confirmation of Purity and Identity

The tentative identification of compounds using HPLC and LC-MS was further analysed via nuclear magnetic resonance (NMR) [59]. A 600 MHz Bruker AVIII HD spectrometer outfitted with a 5 mm BBO H and F cryogenic test was implemented to record NMR spectra. The ^1^H NMR and ^13^C NMR spectra were obtained by implementing standard one-dimensional composite pulse sequencing (zgcppr) and the aid of the use of the reverse gated-decoupling pulse sequence (zgig), respectively, and the parameters were set according to a previous description [59].

### 4.17. Statistical Analysis

For all statistical analysis and interpretation of data, GraphPad Prism 5 was used. Data were analysed using the unpaired Student’s *t*-test (nonparametric, with two-tailed *p*-values) and one-way ANOVA with the help of Bonferroni post hoc tests. Values were introduced as the mean ± SEM, and *p* < 0.05 was set as the significant limit.

## 5. Conclusions

In conclusion, the present study provides definitive evidence for the antidiabetic properties of *A. arabica* bark in high-fat-fed (HFF) obese diabetic rats and reveals that this is due to a broad spectrum of pancreatic and extrapancreatic actions. These serve to enhance insulin secretion, promote insulin action and cellular glucose uptake together with retardation of the digestion and absorption of glucose from food. Phytochemicals responsible for β-cell effects include quercetin, kaempferol and catechin. Such herbal remedies based on *A. arabica* might provide a validated, accessible, and useful adjunctive diabetic treatment, especially in the areas that do not have easy access to established therapies. However, further studies are warranted to assess the potential use of *A. arabica* and its marker compounds in the prevention and management of type 2 diabetes.

## Figures and Tables

**Table 1:** Molecular mass and predicted identity of peak samples of *A. arabica* bark obtained from the preparative RP-HPLC via LC-MS analysis.

Peak Samples	Retention Time (min)	Theoretical Molecular Wt. (Da)	Found Molecular Weight (Da)	Predicted Compounds
P_1_	9.7	302.2	301.2	Quercetin
P_2_	19	290.3	289.0	Catechin
P_3_	23	286.2	285.2	Kaempferol
P_4_	29	-	677.2	Unknown
P_5_	32.5	-	496.9	Unknown

Peaks were separated on a Spectra System LC using a Kinetex 5 µm F5 LC column (150 × 4.6 mm^2^) (Phenomenex). The mass-to-charge ratio (m/z) versus peak intensity was determined.

## Data Availability

The data presented in this study are available on request from the corresponding author. The data are not publicly available due to restrictions.

## Supplementary Materials

The following are available online at https://www.mdpi.com/article/10.3390/plants10061190/s1, Figure S1: Dose-dependent effects of various concentrations of (A & B) hot water extract of *A. arabica* bark, (C) peak samples, (D) Quercetin & (E) Kaempferol at 5.6/16.7mM glucose on LDH release from BRIN-BD11 cells.

## Abbreviations

AWERB	Animal Welfare and Ethical Review Board
HWAA	Hot-water extract of *Acacia arabica*
DM	Diabetes mellitus
T2DM	Type 2 diabetes mellitus
STZ	Streptozotocin
GLP-1	Glucagon-like peptide-1
GIP	Glucose-dependent insulinotropic polypeptide
DPP-IV	Dipeptidyl peptidase-IV
LDL	Low-density lipoprotein
HDL	High-density lipoprotein
HFF	High-fat fed
TG	Triglycerides
TFA	Trifluoroacetic acid
AMC	7-amino-4-methylcoumarin
ANOVA	Analysis of variance
IBMX	Isobutylmethylxanthin
