# Supplementary material for: Identification of Multiple Pancreatic and Extra-Pancreatic Pathways Underlying the Glucose-Lowering Actions of Acacia arabica Bark in Type-2 Diabetes and Isolation of Active Phytoconstituents
Source: Plants (Basel). 2021 Jun 11;10(6):1190. doi: 10.3390/plants10061190 (PMC8230611; doi:10.3390/plants10061190)
Supplement: Supplementary file 1 [file plants-10-01190-s001.zip › plants-1235628-supplementary.pdf]

Supplementary Figure 1: Dose-dependent effects of various concentrations of (A & B) hot water extract of *A. arabica* bark, (C) peak samples, (D) Quercetin & (E) Kaempferol at 5.6/16.7mM glucose on LDH release from BRIN-BD11 cells

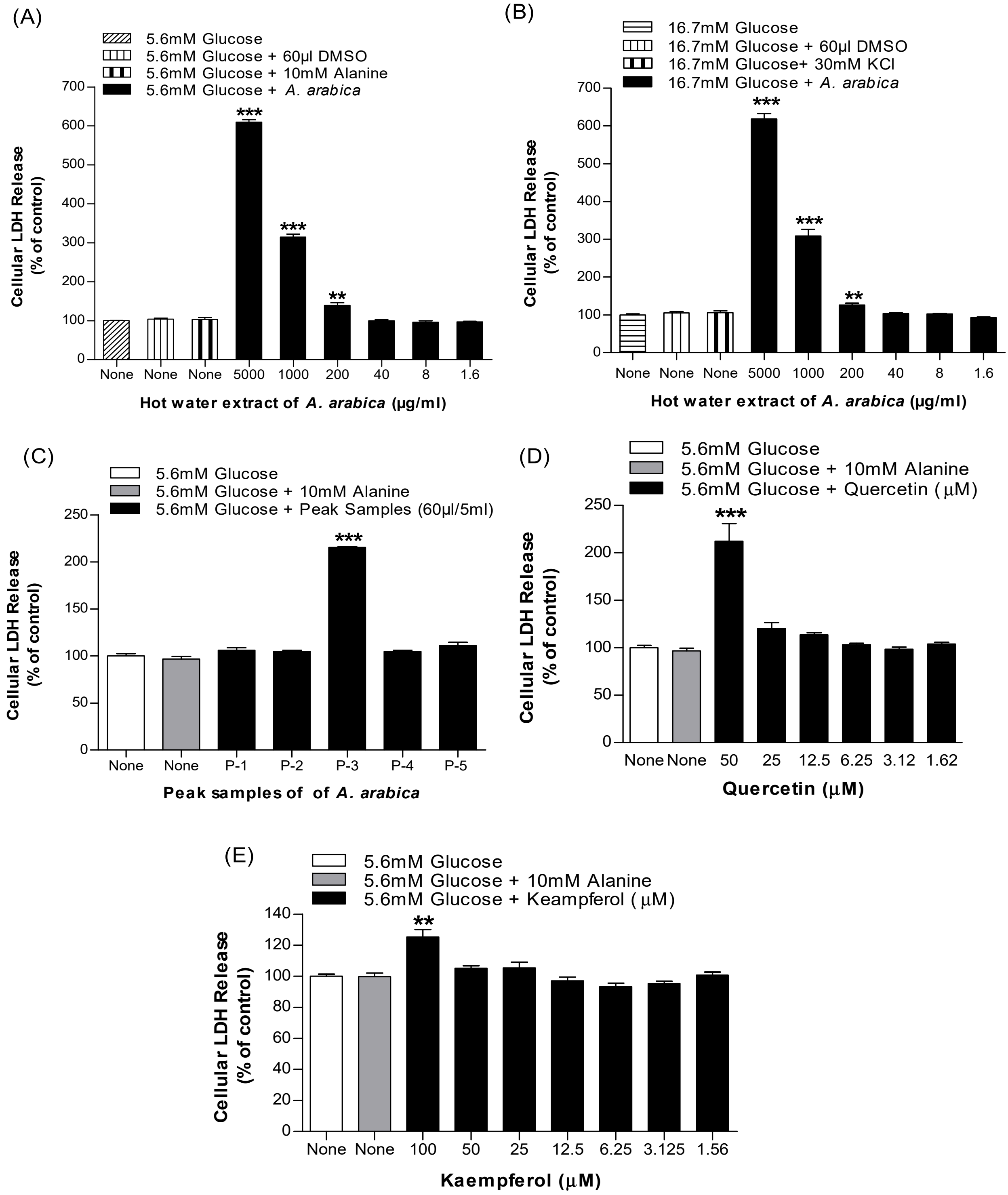

Values are Mean±SEM for n= 4, for LDH release from BRIN BD11 cells. **p<0.01 and ***p<0.001 compared to control (5.6mM glucose alone).
